# Safe Brain Tumor Resection Does not Depend on Surgery Alone - Role of Hemodynamics

**DOI:** 10.1038/s41598-017-05767-2

**Published:** 2017-07-17

**Authors:** Stefanie Bette, Benedikt Wiestler, Felicitas Wiedenmann, Johannes Kaesmacher, Martin Bretschneider, Melanie Barz, Thomas Huber, Yu-Mi Ryang, Eberhard Kochs, Claus Zimmer, Bernhard Meyer, Tobias Boeckh-Behrens, Jan S. Kirschke, Jens Gempt

**Affiliations:** 1 Department of Neuroradiology, Klinikum rechts der Isar, Technische Universität München, Munich, Germany; 2 Department of Neurosurgery, Klinikum rechts der Isar, Technische Universität München, Munich, Germany; 3 Department of Anaesthesiology, Klinikum rechts der Isar, Technische Universität München, Munich, Germany; 4 Institute for Clinical Radiology, Ludwig-Maximilians-Universität München, Munich, Germany

## Abstract

Aim of this study was to determine if perioperative hemodynamics have an impact on perioperative infarct volume and patients’ prognosis. 201 cases with surgery for a newly diagnosed or recurrent glioblastoma were retrospectively analyzed. Clinical data and perioperative hemodynamic parameters, blood tests and time of surgery were recorded. Postoperative infarct volume was quantitatively assessed by semiautomatic segmentation. Mean diastolic blood pressure (dBP) during surgery (rho −0.239, 95% CI −0.11 – −0.367, p = 0.017), liquid balance (rho 0.236, 95% CI 0.1–0.373, p = 0.017) and mean arterial pressure (MAP) during surgery (rho −0.206, 95% CI −0.07 – −0.34, p = 0.041) showed significant correlation to infarct volume. A rank regression model including also age and recurrent surgery as possible confounders revealed mean intraoperative dBP, liquid balance and length of surgery as independent factors for infarct volume. Univariate survival analysis showed mean intraoperative dBP and MAP as significant prognostic factors, length of surgery also remained as significant prognostic factor in a multivariate model. Perioperative close anesthesiologic monitoring of blood pressure and liquid balance is of high significance during brain tumor surgery and should be performed to prevent or minimize perioperative infarctions and to prolong survival.

## Introduction

Perioperative infarction occurs in about 30–80% cases with brain tumor surgery and is associated with postoperative neurological deficits and impaired functional independence^1–4^. Other studies also indicated that postoperative ischemia and postoperatively acquired deficits correlate with overall survival and that perioperative ischemia might introduce hypoxia-mediated tumor growth^5–7^. According to these studies, prevention of perioperative infarction is important for patients’ functional independence and prognosis. Recent studies showed that hypotension during vascular and non-cardiac surgery is a high risk factor for myocardial injury, suggesting that intraoperative monitoring of blood pressure and hemodynamics is of high importance^8, 9^.

For patients undergoing surgery for brain tumors close intraoperative and also postoperative monitoring is performed to avoid excessive variability of blood pressure and heart rate. However, there is no study investigating the correlation of hemodynamic parameters during brain tumor surgery with perioperative infarction and patients’ outcome.

This study therefore aimed to analyze hemodynamic parameters during and after brain tumor surgery and correlate them to postoperative infarct volume and overall survival.

## Patients and Methods

### Patient population

We retrospectively included 179 consecutive patients who underwent 201 surgeries for a newly diagnosed or recurrent glioblastoma (WHO IV) between May 2008 and March 2015. For all patients early postoperative MRI (including diffusion-weighted images) and documentation sheets of pre-/intra- and postoperative hemodynamics were available. The following data were recorded by assessing the patients’ medical charts blinded to the course of hemodynamics: clinical data including past medical history of high blood pressure, diabetes, peripheral arterial occlusive disease (PAOD), previous thromboembolic events as well as smoking, pre-/postoperative Karnofsky Performance Status Scale (KPS), date of initial tumor diagnosis as well as of surgery, previous tumor resections or previous irradiation and date of death or last contact. Two surgeries of the same patient were assessed as independent cases. Only patients with the diagnosis of a glioblastoma (WHO IV) were included in this study; histopathological analysis was performed at the Department of Neuropathology according to the WHO criteria of 2007^11^.

### Anesthesia and Postanesthesia Care Unit

Induction and maintainance of anesthesia were performed according to institutional standards with an infusion of Propofol (usually starting with 5 mg/kg/h) and Remifentanil (starting with 0.2 µg/kg/min). After orotracheal intubation a central venous catheter and an arterial line were established. Dexamethason (in a dose of 40 mg) was given to reduce the tumor-associated edema and mannitol (in a dose of 20 g) to further decrease intracranial pressure.

Lactated Ringers solution was used for fluid resuscitation. In case of larger amounts of blood loss HES 130/0.4 (before June 2013) or Gelafundin 40 (after June 2013) were administered to replace volume loss. After surgery prompt extubation was strived for and the patients were transferred to the post anaesthesia care unit (PACU).

### Hemodynamic Parameters and Monitoring

Prospectively documented data of the hemodynamic parameters before, during and after surgery were collected in each case. Routinely the patients stay overnight at the PACU after surgery. Duration of surgery was recorded for each patient. Blood pressure (BP) was recorded every 5 minutes during surgery and every hour after surgery in the PACU. Further the end tidal CO_2_ (etCO_2_) is recorded during the time of mechanical ventilation every 5 minutes and used for analysis of hypocapnia. For all patients blood tests were performed for: hemoglobin (Hb) and the level of carbondioxide (paCO_2_). For “calibration” of the etCO_2_ (for individual intrapulmonal shunting) the difference from the first corresponding etCO_2_ and paCO_2_ was calculated and therefore documented accordingly to consider the individual arterio-alveolar difference. Volume of fluid in- and output and liquid balance was recorded for each case. Start and duration of surgery as well as the time spent in the PACU were recorded for each case; three time intervals were defined: preparation for surgery (preoperative), surgery (intraoperative), PACU (postoperative). For the three time points the following parameters were assessed respectively: mean and standard deviation (sd) systolic blood pressure (sBP), diastolic BP (dBP), mean arterial pressure (MAP) and paCO_2_. Further the cumulative time of reduced blood pressure (<20% of initial BP) and paCO_2_ (<30 mmHg) was calculated. The minimum Hb was assessed in all patients.

### Magnetic resonance imaging

MRI scans were performed with a 3 Tesla MRI scanner, either Philips Achieva or Philips Ingenia (Philips Medical Systems, The Netherlands B.V.) or Siemens Verio (Siemens Healthcare, Erlangen, Germany). The pre- and postoperative imaging protocols included T2-weighted (w) Fluid Attenuated Inversion Recovery (FLAIR) sequences and T1w sequences with and without contrast agent. The postoperative MRI scans further included diffusion weighted images (DWI).

### Image analysis

Image analysis was performed as described before by a neuroradiologist (SB, 6 years of experience) and a neurosurgeon (JG, 9 years of experience) in consensus, blinded to the hemodynamic parameters^1–3, 5^. Ischemic lesions in the postoperative MRI were defined by a hyperintensity in b-1000 images on DWI and a corresponding hypointensity on apparent diffusion coefficient (ADC) maps. Postoperative changes as well as methemoglobin were excluded by assessing FLAIR images and T1w images without contrast agent as it was also described before^1–3, 5^. Manual segmentation of the postoperative infarct volume was performed by a neuroradiologist, blinded to the hemodynamics and considering the mentioned pitfalls.

For semiautomatic segmentation IPlannet (IPlan 3.0 cranial planning software, Brainlab AG, Feldkirchen, Germany) was used as described before^5^. In addition the volumes of the pre- and postoperative contrast-enhancing tumor part were assessed using manual segmentation.

### Statistical analysis

Statistical analysis was performed using IBM SPSS Statistics Version 23.0 (SPSS Inc., IBM Corp., Armonk, NY, USA). Normally distributed data are displayed as means and standard deviation, non-normally distributed data as median and interquartile range (IR). Overall survival analyses were performed using Kaplan-Meier estimates for univariate analysis and a Cox regression proportional hazards model for multivariate analysis. Dichotomization was performed for median values. For associations between hemodynamic parameters and postoperative infarct volume Fisher’s exact test was used, sensitivity and specificity were calculated for the features (stratified based on the median). Spearman’s correlation coefficient was used to assess the relation between hemodynamics and ischemia volume. To account for type I error inflation, the false discovery rate method proposed by Benjamini and Hochberg was employed. Further, bootstrapping (repeated 1,000 times) was performed to estimate a 95% confidence interval around the correlation coefficient rho. To assess the independent contribution of these parameters to infarct volume, we fitted a rank regression model, considering the non-normality and non-linearity of the dataset, using the “Rfit” package and Wilcoxon scores. Similar to testing the reduction in sums of squares, rank-based regression enables a „drop in dispersion“ test, indicating whether adding parameters to a rank regression model significantly reduces dispersion (improve model fit). All tests were two-sided, assuming a p value < .05 as statistically significant. Calculations were performed in R version 3.2.

## Results

### Patient population

In total, the patient population comprised 179 patients with 201 cases (108 m/71 f; mean age at surgery 61.3y−/+ 12.5) with surgery for a glioblastoma (WHO grade IV); patient characteristics are summarized in Table 1. 135/179 patients presented with initial diagnosis of a glioblastoma, 44/179 patients had recurrent disease and previous surgery. Previous radiotherapy was performed in 42/179 patients. Median KPS was 80 (interquartile range (IQR) 70–90) pre- and postoperatively. Median preoperative tumor volume was 24.6 cm³ (IQR 6.6–51.0 cm³), median postoperative tumor volume 0.0 cm³ (IQR 0.0–1.3 cm³). Arterial hypertension was recorded in 62/179 patients, diabetes in 14/179 patients, previous thromboembolic events in 19/179 patients, peripheral arterial occlusive disease (PAOD) in 2/179 patients. 35/179 patients were smokers. Median overall survival (calculated only for patients with initial tumor diagnosis) was 12.0 months (95% confidence interval (CI) 10.4–13.6).

**Table 1:** Patient characteristics and volumetric measurements.

Variable	Data
Age at date of surgery (n = 179)	61.3 (+/−12.5)
Age at date of initial diagnosis (ID) (n = 179)	61.0 (+/−12.6)
Sex, female	71/179 (39.7%)
Recurrent disease at presentation	44/179 (24.6%)
Radiotherapy before surgery	42/179 (23.5%)
Karnofsky Performance Score (n = 179)	
preoperative	80 (70–90)
postoperative	80 (70–90)
Death during FU (n = 143)	114/143 (79.7%)
OS after ID (n = 143)	12.0 months (95% CI 10.4–13.6)
Arterial hypertension	62/179 (34.6%)
Diabetes	14/179 (7.8%)
Previous thromboembolic events	19/179 (10.6%)
PAOD	2/179 (1.1%)
Smoker	35/179 (19.6%)
Preoperative tumor volume (n = 179)	24.6 cm³ (6.6–51.0 cm³)
Postoperative tumor volume (n = 179)	0.0 cm³ (0.0–1.3 cm³)
Postoperative infarct volume (n = 179)	2.1 cm³ (0.5–6.7 cm³)

normally distributed variables shown as mean +/− standard deviation, non-normally distributed as median (interquartile range).

f, female; m, male; FU: follow up; OS: overall survival, ID: initial diagnosis; PAOD: peripheral arterial occlusive disease.

### Postoperative ischemia

**Figure 1:**
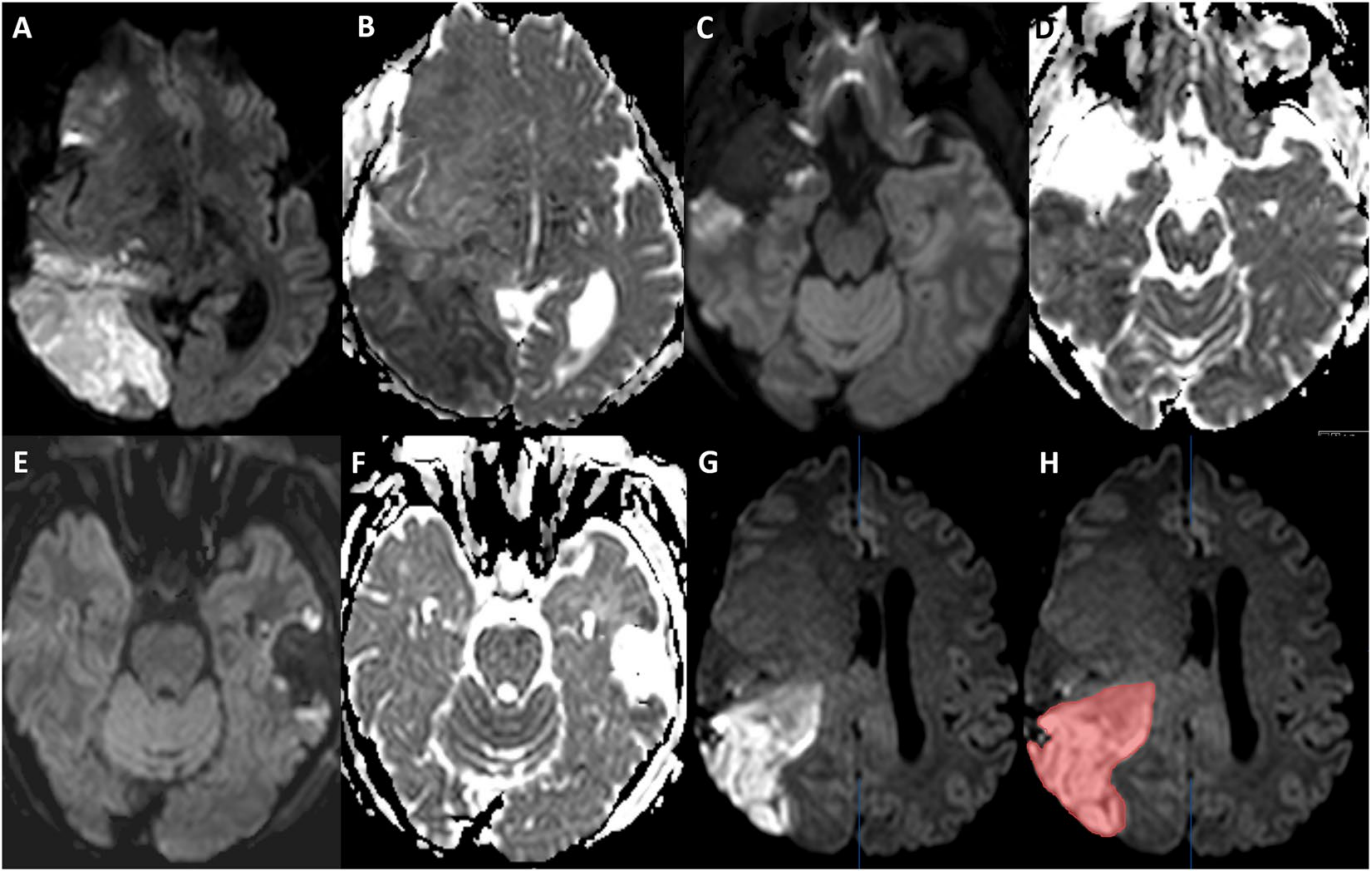
Examples of patients with different infarct volumes. A and B show a large infarction with hyperintensity in b-1000 images (**A**) and corresponding hypointensity in ADC (**B**). (**C** and **D**) show a smaller infarction at the edge of the resection cavity, while E and F show only small ischemic changes. (**G** and **H**) display an example of semiautomatic volumetric measurement of postoperative infarction.

Volumetric measurements of postoperative ischemic changes are summarized in Table 1. In total, 159/179 patients showed ischemic changes on the postoperative MRI. Median infarct volume in all patients (n = 179) was 2.1 cm³ (0.5–6.7 cm³). Figure 1 shows an example of a volumetric measurement of postoperative ischemia.

### Correlations between intra-/postoperative hemodynamics, blood tests and infarct volume

**Figure 2:**
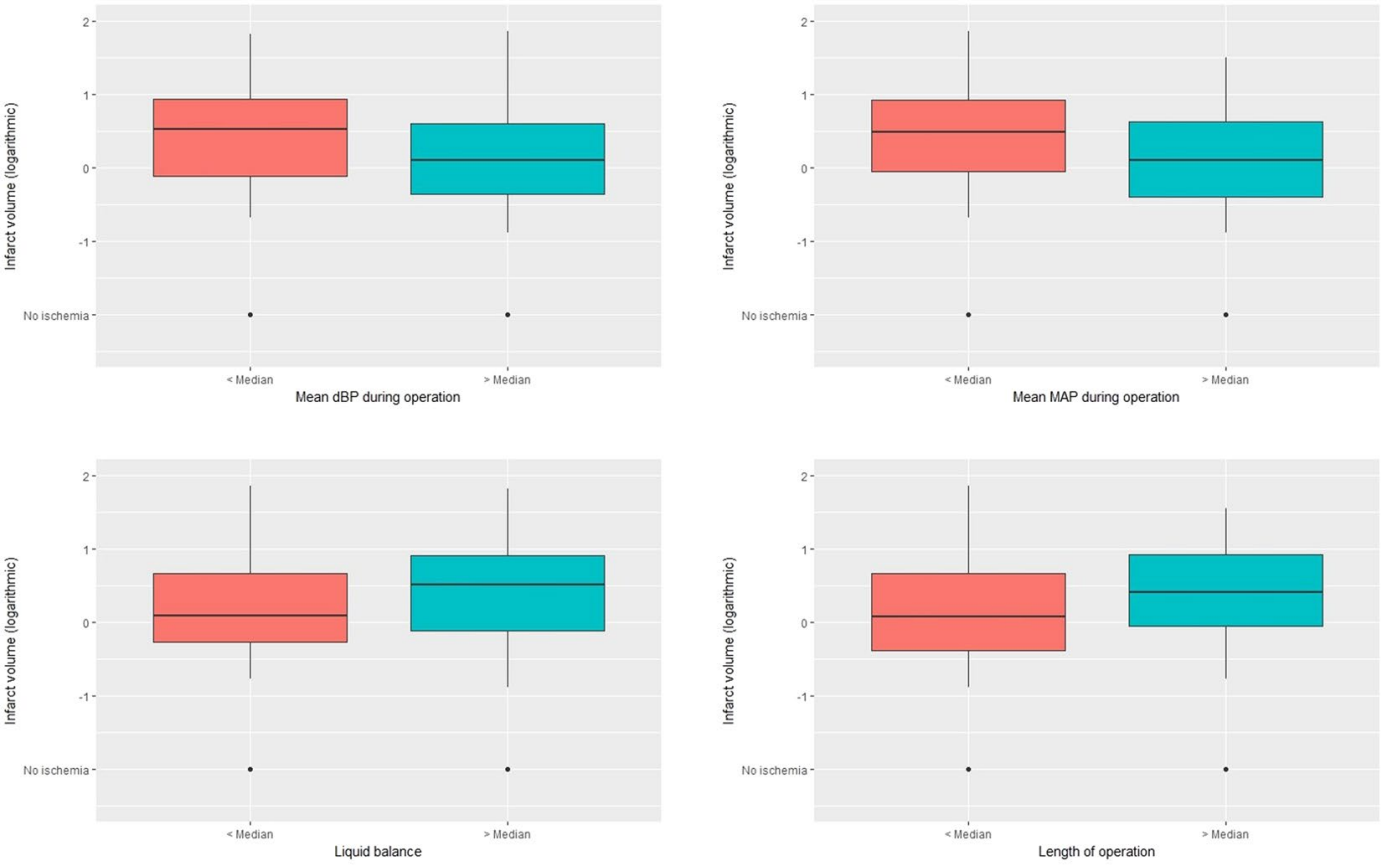
Box plot (dichotomized by the respective median) of mean intraoperative dBP, mean intraoperative MAP, liquid balance & length of surgery and infarct volume. Note that infarct volume (y axis) has been scaled logarithmically.

To assess correlations between hemodynamics and infarct volume, spearman’s correlation coefficient was calculated for all parameters. To adjust for type I error inflation, we performed false-discovery rate correction and bootstrapped each correlation coefficient 1,000 times to construct a 95% confidence interval around the effect estimator. This analysis identified mean diastolic BP during surgery (rho −0.239, 95% CI −0.11 – −0.367, p = 0.017), liquid balance (rho 0.236, 95% CI 0.1–0.373, p = 0.017) and mean arterial pressure during surgery (rho −0.206, 95% CI −0.07 – −0.34, p = 0.041) as significantly associated with infarct volume (Table 2 and Fig. 2). Length of surgery barely missed false discovery rate adjusted significance, with longer durations of surgery being associated with larger infarct volumes (rho 0.183, 95% CI 0.05–0.315, p = 0.081). No significant correlation was observed for the cumulative time of reduced dBP (<20% of the initial BP; rho 0.007, 95% CI −0.13–0.15, p = 0.973) and the cumulative time of reduced paCO_2_ (rho 0.002, 95% CI −0.145–0.15, p = 0.973). Data of all analyzed parameters are summarized in the Supplemental Table 1.

**Table 2:** Spearman’s correlation coefficient rho, its bootstrapped 95% confidence interval and false discover rate-adjusted p value for mean intraoperative dBP, mean intraoperative MAP, liquid balance and length of operation.

Feature	Spearman’s rho	95% Confidence interval	p (FDR-adj.)
Intraoperative mean dBP	−0.238873586	−0.11 – −0.367	0.017024058
Liquid balance	0.236119475	0.1–0.373	0.017024058
Intraoperative mean MAP	−0.20548	−0.07 – −0.34	0.041575504
Length of operation	0.183107007	0.05–0.315	0.081816919

Using Fisher’s exact test significant associations between intraoperative mean dBP (p = 0.023) and postoperative infarct volume as well as between liquid balance (p = 0.015) and infarct volume were observed (Supplemental Table 2).

To assess the independent association of these parameters (intraoperative mean dBP, intraoperative mean MAP, length of surgery and liquid balance) with infarct volume, we employed a rank regression approach, including age at surgery and recurrence as potential confounders. Age at surgery is a known prognostic factor for patients’ outcome after surgery and associated with a higher number of cerebrovascular diseases. Recurrent glioma surgery was shown to be a risk factor for postoperative ischemia due to scar tissue and radiation induced changes^1^.

In this analysis, mean intraoperative dBP, liquid balance and length of surgery were all independently associated with infarct volume, while mean MAP, age and recurrence showed no significant effect on infarct volume (Table 3). For mean MAP, this was not surprising, considering the strong co-linearity with mean dBP, from which it is calculated (Spearman’s rho = 0.91). Consistently, a drop in dispersion test showed a highly significant decrease of dispersion upon incorporation of mean intraoperative dBP, liquid balance of length of surgery into the regression formula (F statistic 8.3912, p = 0.000028737), while the addition of mean intraoperative MAP had no further significant effect (F statistic 2.989612, p = 0.085425).

**Table 3:** Rank regression model for infarct volume as dependent parameter.

Feature	Coefficient	Standard error	p - value
(Intercept)	3.60495996	3.55460617	0.3117934
Intraoperative mean dBP	−0.2806577	0.09642437	0.0040378
Intraoperative mean MAP	0.16467332	0.09324762	0.0790054
Length of surgery	0.01107712	0.00449952	0.014714
Liquid balance	0.00068436	0.000186517	0.0003159
Recurrence, yes vs. no	0.59812028	0.57602504	0.300423
Age	−0.0139845	0.02217059	0.5297171

### Overall survival

**Figure 3:**
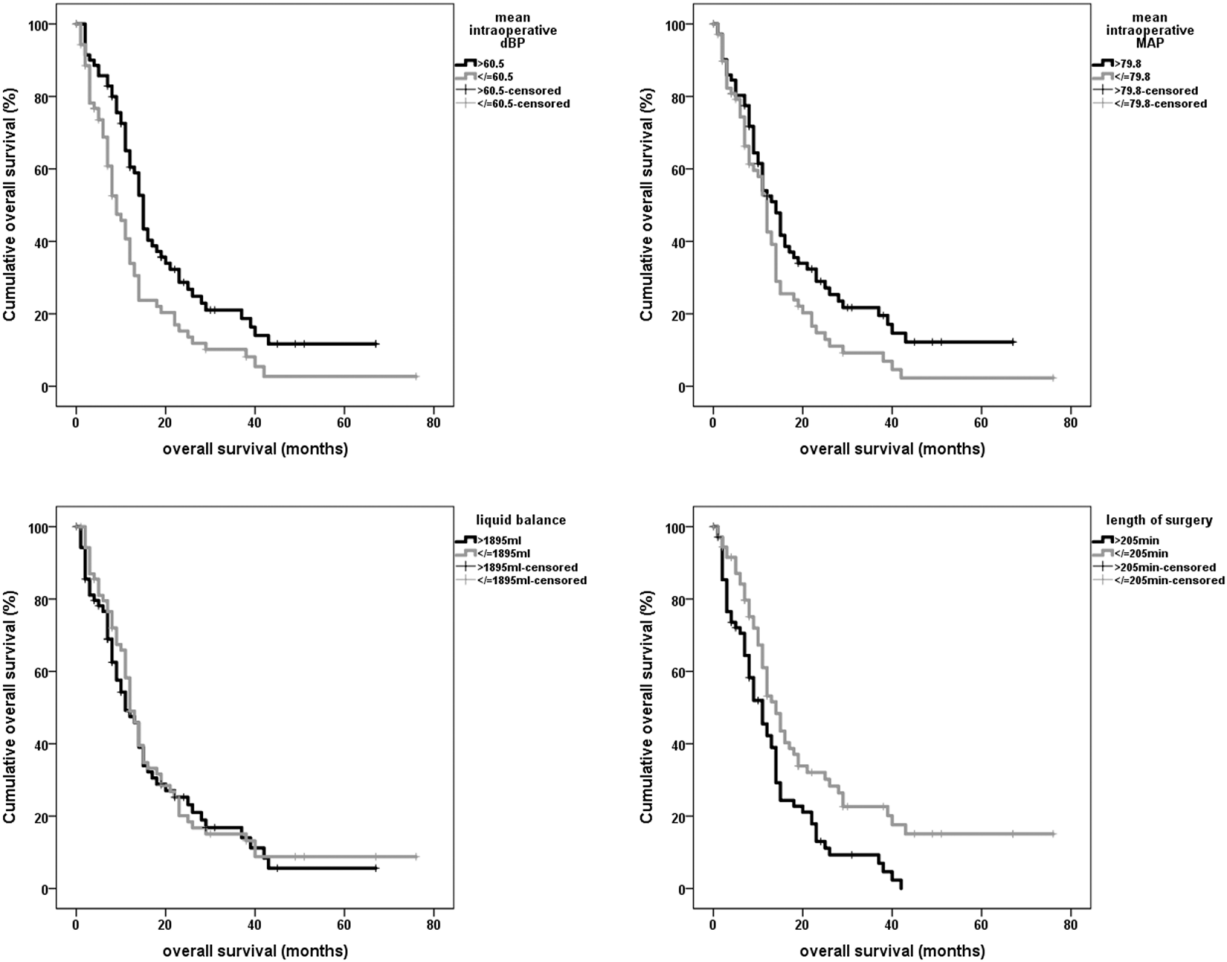
Kaplan Meier estimates for dichotomized mean diastolic blood pressure, MAP, liquid balance and length of surgery.

Univariate and multivariate survival analyses were calculated for cases with initial diagnosis of a glioblastoma. Univariate survival analysis using Kaplan-Meier estimates (log rank) revealed the following parameters as significant prognostic factors: dichotomized mean intraoperative dBP (p = 0.003), mean intraoperative MAP (p = 0.038) and length of surgery (p = 0.002). Liquid balance did not show a significant correlation to survival (p = 0.655) (Fig. 3). Multivariate survival analysis was performed using a Cox regression proportional hazard model with age at initial diagnosis, extent of resection and postoperative KPS (>/=80, <80) as variables. Length of surgery remained as significant prognostic factor in this model (hazard ratio (HR) = 1.003 [95% CI 1.000–1.007], p = 0.027) beneath the known prognostic factors like age (HR = 1.035 [1.016–1.055]), extent of resection (HR = 1.925 [1.251–2.962] for complete resection vs. resection <90%) and postoperative KPS (HR = 2.415 [1.590–3.669]). The multivariate model did not reveal mean intraoperative dBP and MAP as significant prognostic factors.

## Discussion

Mean intraoperative diastolic blood pressure (dBP) during glioblastoma surgery is independently associated with postoperative infarct volume and in univariate analysis also with overall survival suggesting that close monitoring and prevention of low dBP (and hence MAP) is of high significance to prevent or minimize perioperative ischemia and to prolong survival. Further, a high liquid balance and length of surgery were identified as being independently correlated with infarct volume, length of surgery was also shown as an independent prognostic factor for overall survival. These results suggest that apart from neurosurgical techniques tight perioperative blood pressure control is of high importance for patients’ outcome in brain tumor surgery.

To our knowledge this is the first study that assessed the impact of perioperative hemodynamics on infarct volume during brain tumor surgery. There are several studies from other medical specialities – especially cardiovascular surgery - that assessed the influence of intraoperative blood pressure on postoperative outcome. Intraoperative hypotension was described as a risk factor for postoperative myocardial infarction, especially a decrease of >40% of the initial blood pressure and a cumulative time of decreased BP >30 minutes was associated with significantly increased postoperative myocardial injury in a large patient cohort for patients receiving vascular surgery^9^. Another study showed that intraoperative hypotension significantly increased postoperative 30-day mortality^12^. Other studies addressed the influence of intraoperative hypotension on postoperative acute kidney injury^13, 14^. Also the role of blood pressure monitoring during ischemic stroke and during intra-arterial stroke treatment has been investigated by many recent studies^15–18^. These studies also show that low blood pressure or drops of blood pressure are associated with poorer outcome of stroke patients^15–18^.

Our study results are in line with the above-mentioned studies showing that blood pressure is an important prognostic factor, not only in cardiovascular surgery, but also in elective brain tumor surgery with regard to infarct volumes and overall survival.

Mean arterial pressure (MAP) is calculated by diastolic and systolic blood pressure and plays an important role in anesthesiologic blood pressure monitoring. For this calculation dBP shows a higher weighting than sBP. This is also reflected by the results of our study: Whereas in univariate analysis, both dBP and MAP are significantly associated with infarct volume, sBP is not. Multivariate analysis only reveals dBP as significant factor. The results imply that sBP has no relevant association with infarct volume, while monitoring of dBP seems most important. Further studies that investigate the pathomechanisms which cause ischemia during brain surgery will have to be performed.

In our study only the mean dBP, but not the cumulative time of reduced dBP or the dBP variability (standard deviation) had an impact on infarct volume. These results differ from a previous study that found frequency and duration of MAP decrease to be important factors for perioperative myocardial injury^9^. This seeming discrepancy might be explained as in our cohort, blood pressure variability was generally rather low and hence mean BP more important for ischemia development than cumulative time of >20% reduced blood pressure. Further, intrinsic differences in perioperative ischemia mechanisms between brain and heart might contribute to this.

Hypocapnia causes cerebral vasoconstriction and is known as a poor prognostic factor during subarachnoidal hemorrhage (aSAH) and ischemic stroke^19, 20^; during elective brain tumor surgery this has to our knowledge not yet been assessed. In this study no correlation between hypocapnia and cerebral infarctions was shown. This might be explained by the fact that during elective surgery close anesthesiologic monitoring prevented hypo- and hypercapnia and consequently none of the analyzed patients showed severe changes in paCO_2_.

Besides blood pressure perioperative liquid balance was shown as independent factor for perioperative infarctions in our study which to our knowledge has also not been assessed during brain tumor surgery. A previous study showed that a positive liquid balance is associated with poor outcome in patients with aSAH^21^. Also other studies critically scrutinized the effects of hypervolemic therapy for vasospasm after aSAH as only a slight effect on cerebral oxygenation and cerebral blood flow was observed^21–24^. These results are in common with the data of this study as we disclosed a correlation of positive liquid balance to infarct volumes. However the comparison of these results to data of patients with aSAH is difficult since different pathomechanisms occur during these conditions.

Perioperative ischemia during glioma surgery is associated with postoperative neurological deficits, reduced KPS and impaired survival^1, 3, 4, 6^. Other studies also suggested, that perioperative ischemia might introduce hypoxia-mediated invasive tumor growth and is associated with impaired overall survival^5, 7, 25–27^. Therefore the reduction of perioperative infarct volume is of high importance for patients’ outcome. This study presents evidence that intraoperative dBP and MAP are associated with perioperative infarct volume during elective brain tumor surgery and also with overall survival in univariate analysis. Length of surgery was shown as independent prognostic factor for overall survival also in multivariate analysis.

Main limitation of this single-center study is its retrospective design. Notwithstanding the retrospective study design the perioperative hemodynamics have been assessed prospectively in each patient. However the data were not recorded in the framework of a study but at the discretion of the anesthetist in charge which might introduce another unavoidable bias. The anesthesia records are not optimized for scientific use hence they are lacking in resolution regarding the timescale (blood pressure is documented every 5 minutes) and blood pressure scale (discrete lines every 10 mmHg). Further, the semiautomatic segmentation of postoperative infarct volume was performed blinded to the hemodynamic parameters to avoid bias. Further limitations are that other potential prognostic factors for perioperative infarct volume such as vessel anatomy and tumor biology were not considered in this study, and that a few patients were included twice (initial resection and at recurrence). However, we have re-done our analyses including each patient only once (first operation). The results of this analysis were fully concordant with the results presented, making a relevant bias very unlikely.

## Conclusions

Intraoperative mean diastolic blood pressure, liquid balance and length of surgery are all independently associated with perioperative infarct volume during elective brain tumor surgery, underlining the importance of close monitoring of perioperative hemodynamics to avoid or minimize perioperative ischemia. Low mean intraoperative dBP and MAP are associated with impaired overall survival in univariate analysis, length of surgery was shown as an independent factor for overall survival.

### Ethical approval and informed consent

The study was conducted in accordance with the ethical standards of the 1964 Declaration of Helsinki and its later amendments and approved by the local ethics committee (284/15). Informed consent was waived by the local ethics committee for this retrospective non-interventional study.

## Electronic supplementary material

Supplementary Information
