# Supplementary material for: Safe Brain Tumor Resection Does not Depend on Surgery Alone - Role of Hemodynamics
Source: Sci Rep. 2017 Jul 17;7:5585. doi: 10.1038/s41598-017-05767-2 (PMC5514064; doi:10.1038/s41598-017-05767-2)

Safe Brain Tumor Resection Does not Depend on Surgery Alone - Role of Hemodynamics

Stefanie Bette, Benedikt Wiestler, Felicitas Wiedenmann, Johannes Kaesmacher, Martin Bretschneider, Melanie Barz, Thomas Huber, Yu-Mi Ryang, Eberhard Kochs, Claus Zimmer, Bernhard Meyer, Tobias Boeckh-Behrens, Jan S. Kirschke, Jens Gempt

Supplemental Table 1 Bootstrapped spearman's correlation coefficient estimates for all parameters

Feature	rho.bs	rho.bs.se	raw.p	fdr.p
Intraoperative mean dBP	-0.238873586	0.065336572	0.000649033	0.017024058
Liquid balance	0.236119475	0.069775171	0.000920219	0.017024058
Intraoperative mean MAP	-0.205476544	0.068817923	0.003370987	0.041575504
Length of surgery	0.183107007	0.067426821	0.008845072	0.081816919
Minimum Hb	-0.159575334	0.072828597	0.026440392	0.195658904
Diabetes	-0.131803676	0.08304945	0.056986254	0.263561427
Preoperative mean dBP	-0.14028031	0.068435848	0.045799011	0.263561427
Postoperative mean dBP	-0.132483104	0.070376672	0.053009103	0.263561427
Smoker	-0.124098903	0.070413496	0.067594275	0.277887574
Postoperative mean MAP	-0.123964033	0.070389412	0.08497665	0.314413607
Preoperative crit. MAP	0.110668136	0.075229786	0.109475391	0.368235407
PAOD	-0.089852087	0.032211937	0.22223642	0.432776187
Preoperative sd sBP	0.084652975	0.071650171	0.220458455	0.432776187
Preoperative crit. sBP	0.091011491	0.072675889	0.208222392	0.432776187
Preoperative sd dBP	0.095800188	0.065637539	0.167444166	0.432776187
Preoperative mean MAP	-0.099932294	0.066173999	0.166196383	0.432776187
Preoperative sd MAP	0.093215284	0.070013056	0.189396283	0.432776187
Intraoperative mean sBP	-0.099091447	0.069401406	0.16039155	0.432776187
Postoperative mean sBP	-0.096660514	0.072515629	0.175480735	0.432776187
Preoperative crit. dBP	0.077651482	0.074899851	0.257738028	0.476815352
Age	0.068978198	0.069434906	0.340484608	0.599901452
Postoperative crit. dBP	0.047520767	0.070726578	0.489005536	0.808563524
Postoperative crit. MAP	0.043828986	0.072944421	0.502620569	0.808563524
Arterial hypertension	-0.039120786	0.068231518	0.578613166	0.845638847
Thromboembolic event	-0.0355319	0.068057075	0.619795342	0.845638847
Preoperative mean sBP	-0.040314173	0.071524541	0.592027116	0.845638847
Intraoperative sd sBP	0.034503683	0.069487357	0.662798015	0.845638847
Postoperative sd dBP	0.038093988	0.073663976	0.598320676	0.845638847
Postoperative sd MAP	0.03411805	0.072420669	0.655106361	0.845638847
Intraoperative crit. sBP	0,016526007	0.068750519	0.802429962	0.94990122
Intraoperative sd MAP	0.015512395	0.075217489	0.821536191	0.94990122
Postoperative crit. sBP	0.017045708	0.068812775	0.809998239	0.94990122
Intraoperative sd dBP	-0.000629506	0.071516473	0.972685172	0.972685172
Intraoperative crit. dBP	0.00687948	0.072608135	0.935056333	0.972685172
Intraoperative crit. MAP	0.01075946	0.069533842	0.896343854	0.972685172
Intraoperative crit. $etCO_2$	0.002321381	0.075144368	0.961219276	0.972685172
Postoperative sd sBP	0.010783578	0.073021163	0.907673587	0.972685172

rho.bs: bootstrapped rho; rho.bs.se: standard error of bootstrapped rho; raw.p: unadjusted p-value; fdr.p: false discovery rate-adjusted p-value; crit: critical; BP: blood pressure, d: diastolic, s: systolic, sd: standard deviation; MAP: mean arterial pressure, PAOD: peripheral arterial occlusive disease

Supplemental Table 2 Contingency table for postoperative infarct volume

Feature	P-value	Sensitivity (95% CI)	Specificity (95% CI)
Intraoperative mean dBP*	0.023	61.4% (50.4-71.6)	55.8% (46.1-65.1)
Intraoperative mean MAP	0.088	59.1% (48.1-69.5)	54.0% (44.4-63.3)
Liquid balance*	0.015	60.9% (49.9-71.2)	56.9% (47.1-66.3)
Length of surgery	0.256	48.9% (38.1-59.8)	59.2% (49.7-68.4)

All features were stratified for the median (≤ vs. >); *p < 0.05 (Fisher's exact test), CI: confidence interval

Supplementary Figure 1: Scatter plots of mean intraoperative dBP, mean intraoperative MAP, liquid balance & length of surgery and infarct volume. For illustration, a regression line resulting from a univariate rank regression has been plotted (with 95% CI).

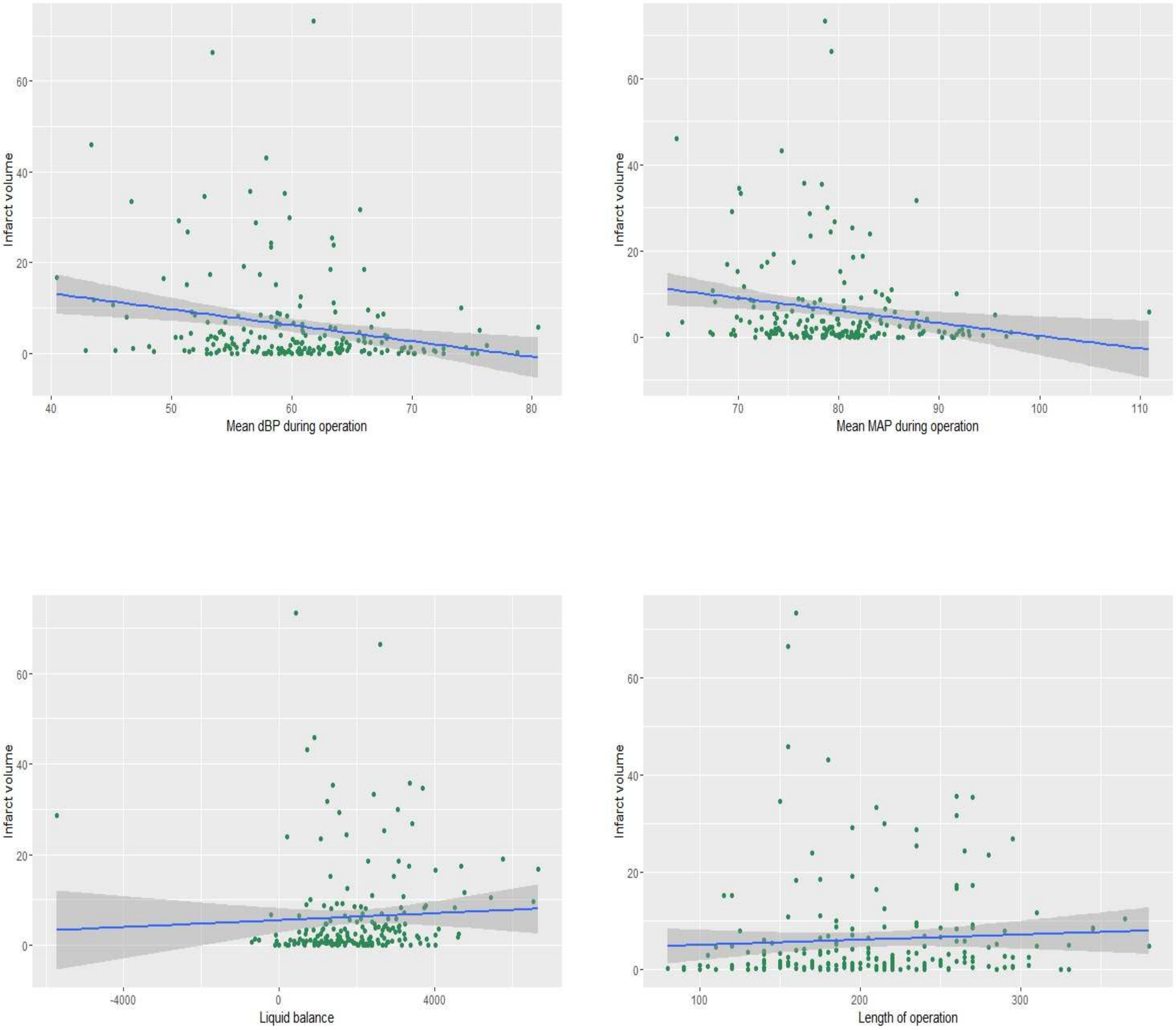

Supplement: Supplementary file 1 — Supplementary Information [file 41598_2017_5767_MOESM1_ESM.pdf]
